# Experimental test of local observer independence

**DOI:** 10.1126/sciadv.aaw9832

**Published:** 2019-09-20

**Authors:** Massimiliano Proietti, Alexander Pickston, Francesco Graffitti, Peter Barrow, Dmytro Kundys, Cyril Branciard, Martin Ringbauer, Alessandro Fedrizzi

**Affiliations:** 1 Scottish Universities Physics Alliance, Institute of Photonics and Quantum Sciences, School of Engineering and Physical Sciences, Heriot-Watt University, Edinburgh EH14 4AS, UK.; 2 Université Grenoble Alpes, CNRS, Grenoble INP, Institut Néel, 38000 Grenoble, France.; 3 Institut für Experimentalphysik, Universität Innsbruck, 6020 Innsbruck, Austria.

## Abstract

The scientific method relies on facts, established through repeated measurements and agreed upon universally, independently of who observed them. In quantum mechanics the objectivity of observations is not so clear, most markedly exposed in Wigner’s eponymous thought experiment where two observers can experience seemingly different realities. The question whether the observers’ narratives can be reconciled has only recently been made accessible to empirical investigation, through recent no-go theorems that construct an extended Wigner’s friend scenario with four observers. In a state-of-the-art six-photon experiment, we realize this extended Wigner’s friend scenario, experimentally violating the associated Bell-type inequality by five standard deviations. If one holds fast to the assumptions of locality and free choice, this result implies that quantum theory should be interpreted in an observer-dependent way.

## Teaser

In the quantum domain, there may not be an objective story to tell.

## INTRODUCTION

The observer’s role as final arbiter of universal facts (*1*) was imperiled by the advent of 20th century science. In relativity, previously absolute observations are now relative to moving reference frames; in quantum theory, all physical processes are continuous and deterministic, except for observations, which are proclaimed to be instantaneous and probabilistic. This fundamental conflict in quantum theory is known as the measurement problem, and it originates because the theory does not provide a precise cut between a process being a measurement or just another unitary physical interaction.

**Fig. 1:**
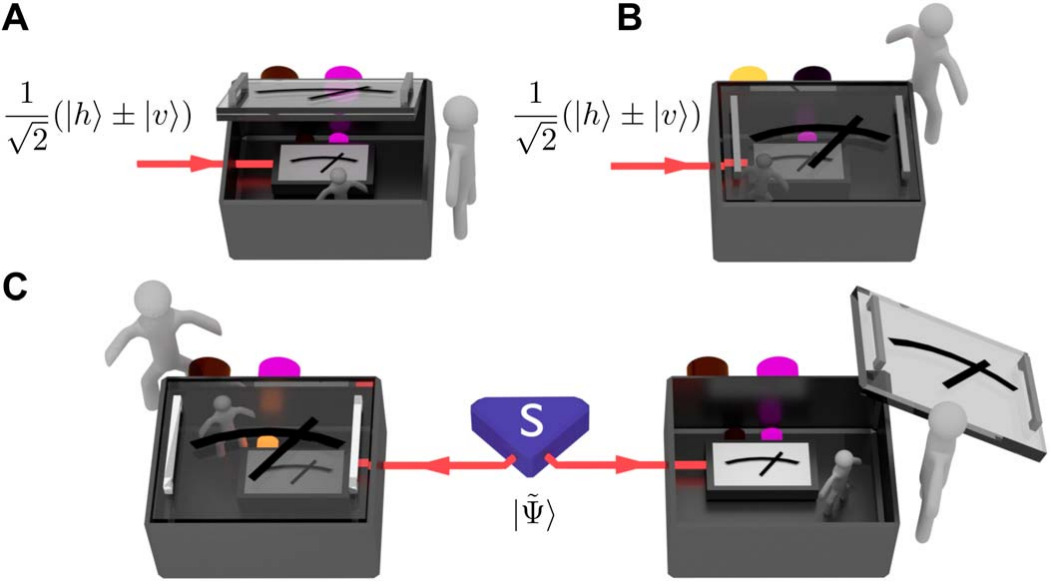
Wigner’s friend experiment. (**A**) A quantum system in an equal superposition of two possible states is measured by Wigner’s friend (inside the box). According to quantum theory, in each run, she will randomly obtain one of two possible measurement outcomes. This can be verified by directly looking into her laboratory and reading which result she recorded. (**B**) From outside the closed laboratory, however, Wigner must describe his friend and her quantum system as a joint entangled state. Wigner can also verify this state assignment through an interference experiment, concluding that his friend cannot have seen a definite outcome in the first place. (**C**) We consider an extended version of that experiment, where an entangled state is sent to two different laboratories, each involving an experimenter and their friend.

This is best illustrated in the seminal “Wigner’s friend” thought experiment (*2*), whose far-reaching implications are only starting to become clear (*3*–*5*). Consider a single photon in a superposition of horizontal ∣*h*〉 and vertical polarization ∣*v*〉, measured in the {∣*h*〉, ∣*v*〉}-basis by an observer—Wigner’s friend—in an isolated laboratory (see Fig. 1, A and B). According to quantum theory, the friend randomly observes one of two possible outcomes in every run of the experiment. The friend’s record, *h* or *v*, can be stored in one of two possible orthogonal states of some physical memory, labeled either ∣“photon is *h* ”〉 or ∣“photon is *v*”〉, and constitutes a “fact” from the friend’s point of view. Wigner, who observes the isolated laboratory from the outside, has no information about his friend’s measurement outcome. According to quantum theory, Wigner must describe the friend’s measurement as a unitary interaction that leaves the photon and the friend’s record in the entangled state (with implicit tensor products)

$$\frac{1}{\sqrt{2}}(|h\rangle \pm |v\rangle )\to \frac{1}{\sqrt{2}}(|h\rangle |“\text{photon is}\;h”\rangle \pm |v\rangle |“\text{photon is}\;v”\rangle )≕|\Phi_{\text{photon}/\text{record}}^{\pm }\rangle$$ (1)

Wigner can now perform an interference experiment in an entangled basis containing the states of Eq. 1 to verify that the photon and his friend’s record are indeed in a superposition—a fact from his point of view. From this fact, Wigner concludes that his friend cannot have recorded a definite outcome. Concurrently, however, the friend does always record a definite outcome, which suggests that the original superposition was destroyed and Wigner should not observe any interference. The friend can even tell Wigner that she recorded a definite outcome (without revealing the result), yet Wigner and his friend’s respective descriptions remain unchanged (*6*). This calls into question the objective status of the facts established by the two observers. Can one reconcile their different records, or are they fundamentally incompatible, so that they cannot be considered objective, observer-independent “facts of the world” (*3*, *4*)?

It was recently shown (*4*) that this question can be addressed formally, by considering an extension of the Wigner’s friend scenario as follows. Consider a pair of physical systems, shared between two separate laboratories controlled by Alice and Bob, respectively (see Fig. 1C). Inside these laboratories, Alice’s friend and Bob’s friend measure their respective system nondestructively and record the outcomes in some memory. Outside these laboratories, in each run of the experiment, Alice and Bob can choose to either measure the state of their friend’s record—i.e., to attest the facts established by their friend, and whose results define the random variables *A*_0_ (for Alice’s friend) and *B*_0_ (for Bob’s friend), or to jointly measure the friend’s record and the system held by the friend—to establish their own facts, defining variables *A*_1_ (for Alice) and *B*_1_ (for Bob). After comparing their results, Alice and Bob can estimate the probability distributions *P*(*A_x_*, *B_y_*) for all four combinations of *x*, *y* = 0,1. As in the original Wigner’s friend Gedankenexperiment, the facts *A*_1_, *B*_1_ attributed to Alice and Bob and *A*_0_, *B*_0_ attributed to their friends’ measurements may be inconsistent.

This raises the question whether a more general framework exists in which all observers can reconcile their recorded facts. We shall call this assumption O, observer-independent facts, stating that a record or piece of information obtained from a measurement should be a fact of the world that all observers can agree on—and that such facts take definite values even if not all are “co-measured” (*7*, *8*). Under the additional assumptions of locality (L)—Alice’s and Bob’s choices do not influence each others’ outcome, and free choice (F)—Alice and Bob can freely choose their measurements *A*_0_, *A*_1_ and *B*_0_, *B*_1_, it should then be possible to construct a single probability distribution *P*(*A*_0_, *A*_1_, *B*_0_, *B*_1_) for the four individual facts under consideration, whose marginals match the probabilities *P*(*A_x_*, *B_y_*) (*3*, *4*).

Any joint probability distribution satisfying these assumptions must then satisfy Bell inequalities (*9*). More specifically, when the variables *A_x_*, *B_y_* take values *a*, *b* ∈ { − 1, + 1}, then the average values 〈*A_x_B_y_*〉 = ∑_*a*,*b*_*abP*(*A_x_* = *a*, *B_y_* = *b*) must obey the Clauser-Horne-Shimony-Holt inequality (*10*)

$$S=\langle A_{1}B_{1}\rangle +\langle A_{1}B_{0}\rangle +\langle A_{0}B_{1}\rangle -\langle A_{0}B_{0}\rangle \leq 2$$ (2)

As shown in (*3*, *4*), a violation of the inequality above is, however, possible in a physical world described by quantum theory. Such a violation would demonstrate that the observed probability distributions *P*(*A_x_*, *B_y_*) are incompatible with assumptions F, L, and O. Therefore, if we accept F and L, it follows that the pieces of information corresponding to facts established by Alice, Bob, and their friends cannot coexist within a single, observer-independent framework (*3*, *4*). Notably, this is the case, even though Alice and Bob can acknowledge the occurrence of a definite outcome in their friend’s closed laboratory.

We note that, although Bell’s mathematical machinery (*11*) is used to show the result, the set of assumptions considered here—and therefore the conclusions that can be drawn from a violation of inequality (*2*)—is different from those in standard Bell tests. While they share assumptions L and F, the third assumption of predetermination (PD) in the original Bell theorem (*12*), differs from our assumption O in that it is only concerned with the deterministic (or otherwise) nature of measurement outcomes, not with their objectivity as in O. A Bell test is indifferent to both the observables used and the underlying system, such that any violation suffices to rule out the conjunction of L, F, and PD. In contrast, a Bell-Wigner test is based on very specific observables that satisfy the definition of an observation given below and thus represent facts relative to different observers. Formally, any Bell-Wigner violation implies a Bell-violation, but not the other way round.

Before we describe our experiment in which we test and indeed violate inequality (*2*), let us first clarify our notion of an observer. Formally, an observation is the act of extracting and storing information about an observed system. Accordingly, we define an observer as any physical system that can extract information from another system by means of some interaction and store that information in a physical memory.

Such an observer can establish facts, to which we assign the value recorded in their memory. Notably, the formalism of quantum mechanics does not make a distinction between large (even conscious) and small physical systems, which is sometimes referred to as universality. Hence, our definition covers human observers, as well as more commonly used nonconscious observers such as (classical or quantum) computers and other measurement devices—even the simplest possible ones, as long as they satisfy the above requirements. We note that the no-go theorem formulated in (*5*) requires observers to be “agents,” who “use” quantum theory to make predictions based on the measurement outcomes. In contrast, for the no-go theorem we tested here (*4*), it is sufficient that they perform a measurement and record the outcome. The enhanced capabilities required of agents were recently discussed in (*13*).

## RESULTS

**Fig. 2:**
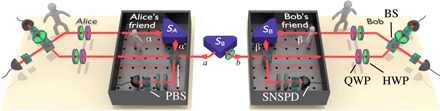
Experimental setup. Pairs of entangled photons from the source *S*_0_, in modes *a* and *b*, respectively, are distributed to Alice’s and Bob’s friend, who locally measure their respective photon in the {∣h〉, ∣v〉}-basis using entangled sources *S_A_*, *S_B_* and type I fusion gates. These use nonclassical interference on a polarizing beam splitter (PBS) together with a set of half-wave (HWP) and quarter-wave plates (QWP). The photons in modes α′ and β′ are detected using superconducting nanowire single-photon detectors (SNSPDs) to herald the successful measurement, while the photons in modes α and β record the friends’ measurement results. Alice (Bob) then either performs a Bell-state measurement via nonclassical interference on a 50/50 beam splitter (BS) on modes *a* and α (*b* and β) to measure *A*_1_ (*B*_1_) and establish her (his) own fact or removes the BS to measure *A*_0_ (*B*_0_) to infer the fact recorded by their respective friend (see the Supplementary Materials for details).

Our experiment makes use of three photon-pair sources optimized for brightness and purity (*14*, *15*) based on Sagnac-type (*16*) design *S*_0_, *S_A_*, and *S_B_* (see Fig. 2), which generate pairs of 1550-nm single photons, entangled in the polarization degree of freedom in the state $|\Psi^{-}\rangle =(|h\rangle |v\rangle -|v\rangle |h\rangle )/\sqrt{2}$. We confirmed the almost ideal quality of the prepared states via quantum state tomography, with typical fidelity $F=99.62_{-0.04}^{+0.01}\%$, purity $P=99.34_{-0.09}^{+0.01}\%$, and entanglement as measured by the concurrence $C=99.38_{-0.10}^{+0.02}\%$ (see Materials and Methods for details). The photon pair from source *S*_0_ is rotated to

$$|\tilde{\Psi }\rangle =1⊗U_{\frac{7\pi }{16}}\;|\Psi^{-}\rangle$$ (3)

using a half-wave plate (HWP) at an angle 7π/16, given by $U_{\frac{7\pi }{16}}=\text{cos}(\frac{7\pi }{8})\;\sigma_{z}+\text{sin}(\frac{7\pi }{8})\;\sigma_{x}$ (where 1 is the identity and σ*_z_* and σ*_x_* are the Pauli operators). This state maximizes the violation of inequality (*2*) for our choice of measurement settings (see Eq. 4).

Source *S*_0_ provides the quantum systems on which Alice’s friend and Bob’s friend perform their measurements. Recalling the above definition of an observer, we use the entangled photon pairs from sources *S_A_* and *S_B_* as the physical systems that, through interaction in a type-I fusion gate (*17*, *18*) between modes *a*, α′ and *b*, β′, respectively (see Fig. 2), are able to extract information and thereby establish their own facts. When successful, the fusion gate realizes a nondestructive polarization measurement of a photon from *S*_0_ in the {∣*h*〉, ∣*v*〉}-basis, whose results ∣“photon is *h*”〉 or ∣“photon is *v*”〉 represent the friend’s record. Via the ancillary entanglement, the extracted information is then stored in the polarization state of the other photon from *S_A_* (*S_B_*)—in mode α (β)—that acts as a memory, while the photon in mode α′ (β′) is absorbed in a single-photon counter to herald the success of the measurement (see Materials and Methods for details). Note that this detection could be delayed until the end of the experiment as it carries no information about the measurement outcome, akin to the observer in the box communicating that an observation took place (*3*, *4*). From Alice’s and Bob’s perspective, the yet undetected photons from *S*_0_, *S_A_*, and *S_B_* are now in a joint four-photon entangled state (see Eq. 11 in Materials and Methods).

To test inequality (*2*), Alice and Bob then measure the following observables on their respective joint photon/friend’s record systems

$$\begin{array}{c} A_{0}=B_{0}=1⊗(|“\text{photon is}\;h”\rangle \;\langle “\text{photon is}\;h”|-|“\text{photon is}\;v”\rangle \;\langle “\text{photon is}\;v”|),\\ A_{1}=B_{1}=|\Phi_{\text{photon}/\text{record}}^{+}\rangle \;\langle \Phi_{\text{photon}/\text{record}}^{+}|-|\Phi_{\text{photon}/\text{record}}^{-}\rangle \;\langle \Phi_{\text{photon}/\text{record}}^{-}| \end{array}$$ (4)

(with $|\Phi_{\text{photon}/\text{record}}^{\pm }\rangle$ as defined in Eq. 1). The observables *A*_0_ and *B*_0_ directly unveil the records established by Alice’s and Bob’s friend, respectively. The observables *A*_1_ and *B*_1_, on the other hand, correspond to Alice’s and Bob’s joint measurements on their friend’s photon and record, and define their own facts in the same way as Wigner in the original thought experiment confirms his entangled state assignment.

**Fig. 3:**
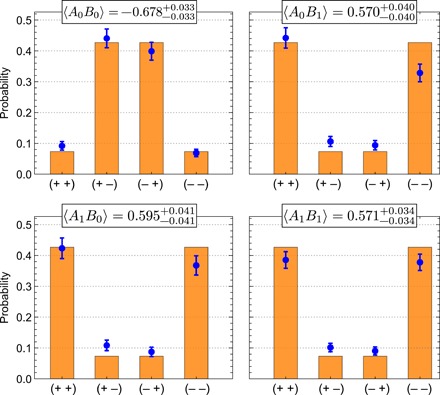
Experimental data. The outcome probabilities comprising each of the four expectation values 〈*A*_0_*B*_0_〉, 〈*A*_0_*B*_1_〉, 〈*A*_1_*B*_0_〉, and 〈*A*_1_*B*_1_〉 are obtained from the measured sixfold coincidence events for each set of 4 × 4 eigenvectors during a fixed time window. Shown here are only the data corresponding to nonzero eigenvalues labeled on the horizontal axes + and − for +1 and −1, respectively, with the full data shown in the Supplementary Materials. The theoretical predictions are shown as orange bars, and each measured expectation value is given above the corresponding subfigure. Uncertainties on the latter and error bars on the data represent 1σ statistical confidence intervals assuming Poissonian counting statistics (see the Supplementary Materials).

We estimate the four average values 〈*A_x_B_y_*〉 in inequality *2* via projection onto each of the 4 × 4 eigenstates of the observables *A_x_* and *B_y_* (see Materials and Methods for details). For the corresponding 64 settings, we collect 1794 six-photon coincidence events over a total measurement time of 360 hours, from which we calculate the probabilities shown in Fig. 3. We achieve a value of $S_{\text{exp}}=2.416_{-0.075}^{+0.075}$, thus violating inequality (*2*) by more than five standard deviations. This result is primarily limited by the higher-order photon emissions from our probabilistic photon sources. Statistical uncertainties are independently estimated using an error propagation approach and a Monte Carlo method. Details are discussed in Materials and Methods.

## DISCUSSION

In principle, “Bell-Wigner tests” like ours are subject to similar loopholes as tests of conventional Bell inequalities (*19*). To address the detection and space-time loopholes, we make the physically reasonable assumption of fair sampling and rely on the empirical absence of signaling between our measurement devices (which we experimentally verified to be in agreement with the expectation from Poissonian statistics). Another loophole may arise if the observables *A*_0_, *B*_0_ that are measured in practice do not strictly correspond to a measurement of the friends’ records. Here, we assume (with reasonable confidence, up to negligible experimental deviations) that the measured observables indeed factorize as in Eq. 4, with the identity on the photon system, so that the above interpretation for *A*_0_, *B*_0_ can be trusted. As discussed in the Supplementary Materials, closing all loopholes in full will be considerably more challenging than for Bell tests.

One might further be tempted to deny our photonic memories the status of “observer.” This, however, would require a convincing revision of our minimal definition of what qualifies as an observer, which typically comes at the cost of introducing new physics that is not described by standard quantum theory. Wigner, for example, argued that the disagreement with his hypothetical friend could not arise due to a supposed impossibility for conscious observers to be in a superposition state (*2*). However, the lack of objectivity revealed by a Bell-Wigner test does not arise in anyone’s consciousness, but between the recorded facts. Because quantum theory does not distinguish between information recorded in a microscopic system (such as our photonic memory) and in a macroscopic system, the conclusions are the same for both: The measurement records are in conflict regardless of the size or complexity of the observer that records them. Implementing the experiment with more complex observers would not necessarily lead to new insights into the specific issue of observer independence in quantum theory. It would, however, serve to show that quantum mechanics still holds at larger scales, ruling out alternative (collapse) models (*20*). However, this is not the point of a Bell-Wigner test—less demanding experiments could show that.

Modulo the potential loopholes and accepting the photons’ status as observers, the violation of inequality (*2*) implies that at least one of the three assumptions of free choice, locality, and observer-independent facts must fail. The related no-go theorem by Frauchiger and Renner (*5*) rests on different assumptions, which do not explicitly include locality. While the precise interpretation of (*5*) within nonlocal theories is under debate (*21*), it seems that abandoning free choice and locality might not resolve the contradiction (*5*). A compelling way to accommodate our result is then to proclaim that facts of the world can only be established by a privileged observer—e.g., one that would have access to the “global wavefunction” in the many worlds interpretation (*22*) or Bohmian mechanics (*23*). Another option is to give up observer independence completely by considering facts only relative to observers (*24*), or by adopting an interpretation such as QBism, where quantum mechanics is just a tool that captures an agent’s subjective prediction of future measurement outcomes (*25*). This choice, however, requires us to embrace the possibility that different observers irreconcilably disagree about what happened in an experiment. A further interesting question is whether the conclusions drawn from Bell or Bell-Wigner tests change under relativistic conditions with non-inertial observers (*26*).

## MATERIALS AND METHODS

### Setup details

A 775-nm, 1.6 ps–pulsed Ti:sapphire laser was focused into a 22-mm periodically poled potassium titanyl phosphate (ppKTP) crystal in a Sagnac-type interferometer (*16*, *27*), where it generated pairs of 1550-nm single photons through collinear type-II parametric down-conversion. The 80-MHz repetition rate of the pump laser was quadrupled through temporal multiplexing (*28*) to suppress higher-order emissions (see fig. S1). We thereby achieved a signal-to-noise ratio (i.e., photon pairs versus higher-order contributions) of 140 ± 10 in each photon source, generating ∼8000 photon pairs mW^−1^ s^−1^ with a typical heralding efficiency $\eta =(\text{cc}/\sqrt{s_{1}s_{2}})$ of ∼50%, where cc is the number of coincidence counts, and *s*_1_ and *s*_2_ are the numbers of singles in the first and second output, respectively. Single photons passed through 3-nm band-pass filters to guarantee high spectral purity and were detected with superconducting nanowire single-photon detectors (SNSPDs) with a detection efficiency of ∼80%. Detector clicks were time-tagged using a field-programmable gate array and processed to detect coincidences within a temporal window of 1 ns.

To benchmark the three required two-qubit states, we performed maximum-likelihood quantum state tomography directly at each source. From the reconstructed density matrices, we computed the fidelity, concurrence, and purity quoted in the main text. Further transmission of the photon pairs to the fusion gates slightly degrades the fidelities of the three entangled pairs to $F_{0}=98.79_{-0.03}^{+0.03}\%$, $F_{A}=98.70_{-0.03}^{+0.03}\%$, and $F_{B}=98.59_{-0.03}^{+0.03}\%$ for sources *S*_0_, *S_A_*, and *S_B_*, respectively (see Fig. 2). This indicates that the optical circuit preserves the excellent quality of the initial states.

### Measurement protocol

We now describe in detail the measurement procedure sketched in Fig. 2. Source *S*_0_ and the HWP on its right output arm produce an entangled pair of photons in the state of Eq. 3. This photon pair is distributed to the laboratories of Alice’s friend and Bob’s friend, who measure their photon using type-I fusion gates (*17*). Each fusion gate is implemented with a polarizing beam splitter (PBS), where horizontally and vertically polarized photons are transmitted and reflected, respectively (by convention collecting a phase *i* for the latter). Two photons entering the PBS from two different inputs with opposite polarization, ∣*h*〉 ∣*v*〉 or ∣*v*〉 ∣ *h*〉, will exit from the same output port and will therefore not lead to coincident detection. Only the coincident ∣*h*〉 ∣*h*〉 and ∣*v*〉 ∣*v*〉 components will be recorded in post-selection. For these post-selected photons, the fusion gate induces the following transformations

$$\begin{array}{c} |h\rangle |h\rangle \overset{\text{PBS}}{\to }|h\rangle |h\rangle \overset{Q/\text{HWP}}{\to }|h\rangle \frac{|h\rangle +i|v\rangle }{\sqrt{2}},\\ |v\rangle |v\rangle \overset{\text{PBS}}{\to }-|v\rangle |v\rangle \overset{Q/\text{HWP}}{\to }-|v\rangle \frac{|h\rangle -i|v\rangle }{\sqrt{2}} \end{array}$$ (5)

where Q/HWP refers to the combination of a quarter-wave plate at π/4 and a half-wave plate at π/8 behind the PBS (see Fig. 2). The second (heralding) photon in the above equation is then projected onto the state ∣*h*〉 via another PBS. The type-I fusion gate thus implements the operation

$${\mathrm{FG}}_{I}=\frac{1}{\sqrt{2}}(|h\rangle \langle h|\langle h|-|v\rangle \langle v|\langle v|)$$ (6)

where the factor $\frac{1}{\sqrt{2}}$ indicates the success probability of the gate of $\frac{1}{2}$.

To use the fusion gate to measure photon *a* (see Fig. 2) nondestructively, Alice’s friend uses an ancilla from the entangled pair created by *S_A_*, prepared as ∣Ψ^−^〉_α′α_. Depending on the state of the incoming photon, the operation performed by Alice’s friend transforms the overall state as

$$\begin{array}{c} |h\rangle {}_{a}|\Psi^{-}\rangle {}_{\alpha \prime \alpha }=\frac{1}{\sqrt{2}}(|h\rangle {}_{a}|h\rangle {}_{\alpha \prime }|v\rangle {}_{\alpha }-|h\rangle {}_{a}|v\rangle {}_{\alpha \prime }|h\rangle {}_{\alpha })\overset{{\mathrm{FG}}_{I}}{\to }\frac{1}{2}|h\rangle {}_{a}|v\rangle {}_{\alpha },\\ |v\rangle {}_{a}|\Psi^{-}\rangle {}_{\alpha \prime \alpha }=\frac{1}{\sqrt{2}}(|v\rangle {}_{a}|h\rangle {}_{\alpha \prime }|v\rangle {}_{\alpha }-|v\rangle {}_{a}|v\rangle {}_{\alpha \prime }|h\rangle {}_{\alpha })\overset{{\mathrm{FG}}_{I}}{\to }\frac{1}{2}|v\rangle {}_{a}|h\rangle {}_{\alpha } \end{array}$$ (7)

Hence, the state ∣*h*〉*_a_* or ∣*v*〉*_a_* of the external photon in mode *a* is copied, after being flipped (*h* ↔ *v*), onto Alice’s friend’s photon in mode α. This corresponds to a measurement of the incoming photon in the {∣*h*〉, ∣*v*〉}-basis, with the outcome being recorded in the state of photon α such that we can write

$$|“\text{photon is}\;h”\rangle {}_{\alpha }=|v\rangle {}_{\alpha },|“\text{photon is}\;v”\rangle {}_{\alpha }=|h\rangle {}_{\alpha }$$ (8)

The amplitudes $\frac{1}{2}$ in Eq. 7 indicate the total success probability of $\frac{1}{4}$ for this procedure.

Consider now the central source *S*_0_ together with Alice’s and Bob’s friend laboratories. According to Eq. 3, the state generated by *S*_0_ is, after the unitary $U_{\frac{7\pi }{16}}$

$$\begin{array}{cc} |\tilde{\Psi }\rangle {}_{\mathrm{ab}}= & \frac{1}{\sqrt{2}}\text{cos}\frac{\pi }{8}\;(|h\rangle {}_{a}|v\rangle {}_{b}+|v\rangle {}_{a}|h\rangle {}_{b})+\\ & \frac{1}{\sqrt{2}}\text{sin}\frac{\pi }{8}\;(|h\rangle {}_{a}|h\rangle {}_{b}-|v\rangle {}_{a}|v\rangle {}_{b}) \end{array}$$ (9)

The transformations induced by Alice’s and Bob’s friend are then, according to Eq. 7

$$|\tilde{\Psi }\rangle {}_{\mathrm{ab}}|\Psi^{-}\rangle {}_{\alpha \prime \alpha }|\Psi^{-}\rangle {}_{\beta \prime \beta }\overset{{\mathrm{FG}}_{I}^{⊗2}}{\to }\frac{1}{4}|\tilde{\Psi }\prime \rangle {}_{a\alpha b\beta }$$ (10)

with a global success probability of $\frac{1}{16}$. The state

$$\begin{array}{cc} |\tilde{\Psi }\prime \rangle {}_{a\alpha b\beta }= & \frac{1}{\sqrt{2}}\text{cos}\frac{\pi }{8}\;(|\mathrm{hv}\rangle {}_{a\alpha }|\mathrm{vh}\rangle {}_{b\beta }+|\mathrm{vh}\rangle {}_{a\alpha }|\mathrm{hv}\rangle {}_{b\beta })+\\ & \frac{1}{\sqrt{2}}\text{sin}\frac{\pi }{8}\;(|\mathrm{hv}\rangle {}_{a\alpha }|\mathrm{hv}\rangle {}_{b\beta }-|\mathrm{vh}\rangle {}_{a\alpha }|\mathrm{vh}\rangle {}_{b\beta }) \end{array}$$ (11)

is the four-photon state shared by Alice and Bob when both fusion gates are successful.

Recalling from Eq. 8 how the friends’ measurement results are encoded in their polarization states, the observables of Eq. 4 to be measured on $|{\tilde{\Psi }}^{\prime }\rangle {}_{a\alpha b\beta }$ are

$$\begin{array}{c} A_{0}=B_{0}=1⊗(|v\rangle \;\langle v|-|h\rangle \;\langle h|),\\ A_{1}=B_{1}=|\Psi^{+}\rangle \;\langle \Psi^{+}|-|\Psi^{-}\rangle \;\langle \Psi^{-}| \end{array}$$ (12)

with $|\Psi^{\pm }\rangle =\frac{1}{\sqrt{2}}(|\mathrm{hv}\rangle \pm |\mathrm{vh}\rangle )$. To obtain 〈*A_x_B_y_*〉, we projected these states onto all combinations of eigenstates of *A_x_* and *B_y_* individually and recorded six-photon coincidence events for a fixed duration. More specifically, to measure *A*_0_ (similarly *B*_0_), we projected onto ∣*hv*〉_*a*α_ and ∣*vv*〉_*a*α_ (eigenvalue +1), and ∣*hh*〉_*a*α_ and ∣*vh*〉_*a*α_ (eigenvalue −1) using QWP and HWP to implement local rotations before the final PBS, not using the beam splitter (BS) in Fig. 2. Note that *A*_0_ cannot be simply measured by ignoring photon *a* due to the probabilistic nature of the photon source. Hence, this photon has to be measured in a polarization-insensitive way, which, due to the polarization-sensitive nature of the photon-detectors, is best achieved by summing over the projections onto both orthogonal polarizations. To measure *A*_1_ (*B*_1_), we used a 50/50 BS followed by projection onto ∣*vh*〉. Because of nonclassical interference in the BS, this implements a projection onto the singlet state ∣Ψ^−^〉_*a*α_ with success probability $\frac{1}{2}$. Using quantum measurement tomography, we verified this Bell-state measurement with a fidelity of $F_{\text{bsm}}=96.84_{-0.05}^{+0.05}$. Projections on the other Bell states are possible via local rotations using the same QWP and HWP as before. Here, ∣Ψ^+^〉_*a*α_ takes eigenvalue +1, ∣Ψ^−^〉_*a*α_ takes eigenvalue −1, and $|\Phi^{\pm }\rangle {}_{a\alpha }=\frac{1}{\sqrt{2}}{\left(|\mathrm{hh}\rangle \pm |\mathrm{vv}\rangle \right)}_{a\alpha }$ takes eigenvalue 0. Probabilities are obtained from normalizing the measured counts with respect to the total of the 16 measurements for each pair of observables (see fig. S2). The theoretically expected values for the various probabilities are $\frac{1}{4}(1+\frac{1}{\sqrt{2}})≃0.427$, $\frac{1}{4}(1+\frac{1}{\sqrt{2}})≃0.073$, or 0. In addition to this result, an alternative measurement protocol for *A*_0_ and *B*_0_ is presented in the Supplementary Materials.

### Error analysis

As described previously, each average value 〈*A_x_B_y_*〉 was calculated from 16 measured sixfold coincidence counts *n_i_*. These numbers follow a Poisson distribution with variance $\sigma_{n_{i}}^{2}=n_{i}$. The uncertainty on 〈*A_x_B_y_*〉 = *f*(*n*_1_, …, *n*_16_) can then be computed using

$$\sigma_{f}^{2}(n_{1},\ldots ,n_{16})=\sum_{i=1}^{16}{\left(\frac{\partial f}{\partial n_{i}}\right)}^{2}\sigma_{n_{i}}^{2}$$ (13)

Because the four averages 〈*A*_1_*B*_1_〉, 〈*A*_1_*B*_0_〉, 〈*A*_0_*B*_1_〉, and 〈*A*_0_*B*_0_〉 are statistically independent, the uncertainties can be calculated independently and combined to estimate the uncertainty on *S*. To take into account potentially asymmetric errors in the limit of small count rates, we computed the uncertainty on the Bell-Wigner parameter *S* using a Monte Carlo routine with 100,000 samples. The values obtained through these two methods agree to within 0.0032.

Note that in the results shown in fig. S3 with the observables of eq. S1, errors are correlated due to normalization with a common total. Accounting for this in the error propagation results in slightly larger statistical uncertainty.

The Bell-Wigner value *S*_exp_ that can be achieved experimentally is primarily limited by multipair emissions from our probabilistic photon pair sources. We first note that any emission of three pairs from any subset of our three sources occurs with roughly similar probability. To exclude unwanted terms, we used six-fold coincidence detection, which can only be successful for an emission of one pair each in *S*_0_, *S_A_*, and *S_B_*, or three pairs in *S*_0_. The latter would amount to noise but is excluded by our cross-polarization design and can thus not lead to a coincidence detection. This leaves higher-order contributions, where at least four photon pairs are produced as the main source of errors. Because such events scale with a higher exponent of the pump power, they are suppressed in our experiment by working with a relatively low pump power of 100 mW.

## Supplementary Material

http://advances.sciencemag.org/cgi/content/full/5/9/eaaw9832/DC1

Download PDF

Experimental test of local observer independence

## SUPPLEMENTARY MATERIALS

Supplementary material for this article is available at http://advances.sciencemag.org/cgi/content/full/5/9/eaaw9832/DC1 Supplementary Text Fig. S1. Detailed experimental setup. Fig. S2. Full experimental data. Fig. S3. Alternative protocol experimental data. References (*29*–*31*)
