# Supplementary material for: Experimental test of local observer independence
Source: Sci Adv. 2019 Sep 20;5(9):eaaw9832. doi: 10.1126/sciadv.aaw9832 (PMC6754223; doi:10.1126/sciadv.aaw9832)
Supplement: http://advances.sciencemag.org/cgi/content/full/5/9/eaaw9832/DC1 [file supp_5_9_eaaw9832__index.html]

Science Advances | Science AdvancesAAASSearchScience AdvancesMenu

## Supplementary Materials

**This PDF file includes:**

- Supplementary Text
- Fig. S1. Detailed experimental setup.
- Fig. S2. Full experimental data.
- Fig. S3. Alternative protocol experimental data.
- References (*29*–*31*)

Download PDF

**Files in this Data Supplement:**

- Adobe PDF - aaw9832\_SM.pdf
