# Supplementary material for: Experimental test of local observer independence
Source: Sci Adv. 2019 Sep 20;5(9):eaaw9832. doi: 10.1126/sciadv.aaw9832 (PMC6754223; doi:10.1126/sciadv.aaw9832)
Supplement: Download PDF [file aaw9832_SM.pdf]

Science Advances

AAAS

advances.sciencemag.org/cgi/content/full/5/9/eaaw9832/DC1

Supplementary Materials for

Experimental test of local observer independence

Massimiliano Proietti, Alexander Pickston, Francesco Graffitti, Peter Barrow, Dmytro Kundys, Cyril Branciard, Martin Ringbauer, Alessandro Fedrizzi*

*Corresponding author. Email: a.fedrizzi@hw.ac.uk

Published 20 September 2019, *Sci. Adv.* **5**, eaaw9832 (2019)
DOI: 10.1126/sciadv.aaw9832

This PDF file includes:

Supplementary Text
Fig. S1. Detailed experimental setup.
Fig. S2. Full experimental data.
Fig. S3. Alternative protocol experimental data.
References (*29*–*31*)

Supplementary Text

Towards a loophole-free "Bell-Wigner" test. Since our experiment relies on the same assumptions as traditional Bell tests, it is subject to the same conceptual and technical loopholes: locality, freedom of choice and the detection loophole. Due to the increased complexity of our experiment, compared to a standard Bell test, the practical requirements for closing these loopholes are significantly more challenging. We now briefly discuss how these loopholes could be closed in the future.

The configuration of our experiment makes it analogous to an "event-ready" Bell test, where the detection of the ancilla photons in the fusion gates heralds which events should be kept for the Bell-Wigner test. In such a configuration, closing the locality and freedom of choice loopholes requires the heralding events to be space-like separated from Alice and Bob's setting choices, which should each be space-like separated from the measurement outcome of the other party. This imposes stringent space-time location requirements for a Bell-Wigner test closing these loopholes.

The detection loophole arises because only a fraction of all created photons is detected. In our "event-ready" configuration, the limited success probability of the fusion gates is not an issue: only heralded events will contribute to the Bell-Wigner test. Nevertheless, to ensure that the fusion gates are indeed event-ready, the ancilla detectors should be photon-number-resolving.

To measure the observables A_x, B_y, we chose to project the photon states onto their different eigenstates separately. To close the detection loophole one cannot follow such an approach: the measurement protocol should be able to project the states onto any of the eigenstates in any run of the experiment.

To measure A_0/B_0 from Eq.(4), one could pass the friends' photon through a PBS, with detectors at both outputs. As for A_1/B_1, a full Bell-state measurement (which is impossible with linear quantum optics (29)) is not required: it suffices to distinguish $|\Psi^+\rangle$, $|\Psi^-\rangle$, and have a third outcome for $|\Phi^\pm\rangle$ (see Eq.(12)). This can be realised with a small modification to our setup, with detectors added on the second outputs of Alice and Bob's PBS (30). An even simpler measurement would discriminate e.g. $|\Psi^-\rangle$ from the other three Bell states, thus measuring the observables $A_1 = B_1 = \mathbb{1} - 2|\Psi^-\rangle\langle\Psi^-|$; this would not change anything in an ideal implementation, but simplifies the analysis with detection inefficiencies below.

Even the best photon detectors aren't 100% efficient and optical loss is unavoidable. Assuming a symmetric combined detection efficiency per photon of η, the measurement of A_0/B_0 requires one detector to click and would succeed with probability η, while the measurement of A_1/B_1 requires two detectors to fire and would work as expected with probability η^2. When a detector fails to click, a simple strategy is to output a fixed pre-defined value for the measurement outcome, e.g. +1. Then, for Eq.(11) the average values $\langle A_x B_y\rangle$ are theoretically expected to be $\langle A_0 B_0\rangle = \eta^2(-\frac{1}{\sqrt{2}}) + (1-\eta)^2$, $\langle A_0 B_1\rangle = \langle A_1 B_0\rangle = \eta^3\frac{1}{\sqrt{2}} + (1-\eta)(1-\eta^2)$ and $\langle A_1 B_1\rangle = \eta^4\frac{1}{\sqrt{2}} + (1-\eta^2)^2$. With these values, the minimal required detection efficiency to violate inequality (2) with (unrealistically) perfect quantum states and measurements

is $\eta > 2\sqrt{3(1-\frac{1}{\sqrt{2}})} - 1 \simeq 0.875$. This is a more stringent requirement than for a standard test of the CHSH inequality, for which a similar analysis for maximally entangled states yields $\eta > 2\sqrt{2} - 2 \simeq 0.828$. To relax this requirement, one might attempt similar tricks as for standard Bell tests, e.g. to use non-maximally entangled states (31), although this will come at the cost of a reduced violation of the inequality.

Note, finally, that in the conclusions we draw from the violation of inequality (2), we need to trust that A_0 and B_0 indeed directly measure the memory of Alice and Bob's friends, so as to unveil their respective facts. A new loophole may be open, now specific to Bell-Wigner tests, if such an interpretation cannot be maintained. To address this loophole with a setup like ours, one should use measurement devices for A_0 and B_0 that clearly separate the initial systems and the memories of each friend, and only "looks" at the memory photons, rather than at the system photon + memory photon together; we also leave this possibility as a challenge for future Bell-Wigner experimental tests.

Alternative observables A_0, B_0. In Ref.(4) the observables A_0, B_0 were defined as

$$A_0 = B_0 = |h\rangle\langle h| \otimes |\text{“photon is } h\text{”}\rangle\langle\text{“photon is } h\text{”}| - |v\rangle\langle v| \otimes |\text{“photon is } v\text{”}\rangle\langle\text{“photon is } v\text{”}| \quad \text{(S1)}$$

which have a slightly different physical interpretation. The observables used in the main text and defined in Eq.(4) directly measure the facts established by the friend, as recorded in their memory. In contrast, the observables in Eq.(S1) can be understood as not only a measurement of the friend's record (to establish a "fact for the friend"), but also of the original photon measured by the friend, as a consistency check: if the state of the photon is found to be inconsistent with the friend's record, the definition above assigns a value 0 for the measurement result.

Our experiment also allows us to test inequality (2) using this alternative definition of A_0, B_0. Indeed, from the experimental data shown in fig S2, it suffices (according to Eq. (S1) and recalling Eq.(8)) to assign the eigenstate/eigenvalue according to $|hv\rangle \to +1$, $|vh\rangle \to -1$ and $|hh\rangle, |vv\rangle \to 0$ in the calculation of the average values $\langle A_x B_y\rangle$. We thus obtain the three average values $\langle A_0 B_0\rangle = 0.662^{+0.033}_{-0.033}$, $\langle A_0 B_1\rangle = 0.573^{+0.039}_{-0.039}$ and $\langle A_1 B_0\rangle = 0.600^{+0.040}_{-0.040}$ with $\langle A_1 B_1\rangle$ unchanged. With these values, we have $S_{exp} = 2.407^{+0.073}_{-0.073}$, again violating inequality (2) by more than 5 standard deviations. As in the main text, errors are computed assuming Poissonian photon counting statistics, see below for details.

Alternative measurement protocol for A_0, B_0. Recall that in order to measure A_0 (similarly B_0), the beam splitter for Alice in Fig.(2) has to be removed compared to the measurement of A_1. A less invasive method (which does not compromise the alignment of our optical elements) is to introduce linear polarisers in modes $a(b)$ and $\alpha(\beta)$. This effectively measures the photons before the BS, preventing interference.

We implemented this procedure for the alternative definition of A_0 and B_0 in Eq. (S1). Since this approach leads to a reduced success probability of the measurement of $A_0(B_0)$ by a factor

$1/4$, we measured all 16 eigenvectors only for $\langle A_1 B_1 \rangle$. For the other observables we measured the eigenvectors with non-zero eigenvalues and normalised all data with respect to the total counts for $\langle A_1 B_1 \rangle$, fig. S3. This slightly increases experimental uncertainties, which we have taken into account in our error analysis. The expectation values so obtained are $\langle A_0 B_0 \rangle = 0.609^{+0.048}_{-0.048}$, $\langle A_0 B_1 \rangle = 0.577^{+0.049}_{-0.049}$ and $\langle A_1 B_0 \rangle = 0.588^{+0.049}_{-0.049}$ with $\langle A_1 B_1 \rangle$ unchanged, and $S_{exp} = 2.346^{+0.110}_{-0.110}$, violating the Bell-Wigner inequality by more than 3 standard deviations. We note that the violation observed with this method is somewhat reduced because of $\sim 4.83 \pm 0.97\%$ loss that is introduced by the polarisers. This effectively reduces the number of counts that are observed in the settings A_0 and B_0 compared to the normalisation used, and thereby reduces the expectation values $\langle A_0 B_1 \rangle$ and $\langle A_1 B_0 \rangle$, and $\langle A_0 B_0 \rangle$, leading to a reduced violation.

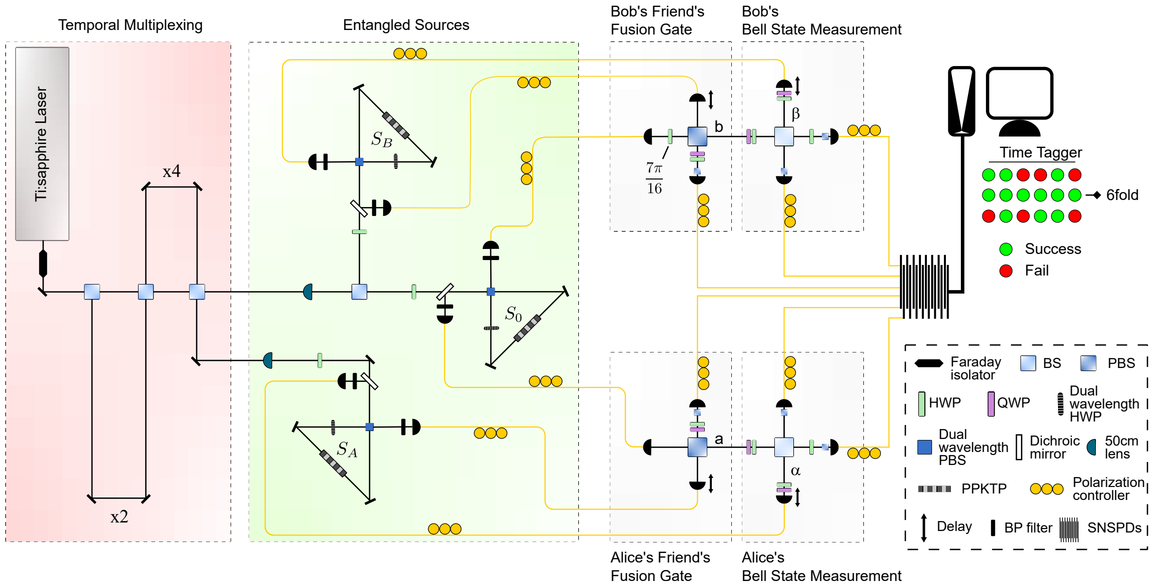

Fig. S1. Detailed experimental setup. The Ti:sapphire laser beam is protected from back-reflections by a Faraday isolator and spatially filtered using a short single-mode fibre (not shown). The laser beam is then temporally multiplexed to effectively quadruple the pulse rate. The pump is then delivered to three Sagnac-interferometer sources to create polarisation entangled photon pairs. The outputs of each source are coupled to single-mode fibres and delivered to the measurement stages. Fibre polarisation controllers are used to maintain the polarisation states of the photons during transport. The three entangled pairs are then subject to two fusion gates, where temporal mode matching is achieved by employing physical delays as indicated. One photon at each measurement stage acts as a heralding signal for the success of the fusion gate, while the other two are subject to a Bell-state measurement on a 50/50 beam splitter, or to a direct measurement without the BS (for A_0, B_0), followed by projection onto orthogonal polarisations. Finally, all six photons are fibre-coupled and detected by the SNSPDs whose detection is processed by a classical computer to find 6-photon coincidence events.

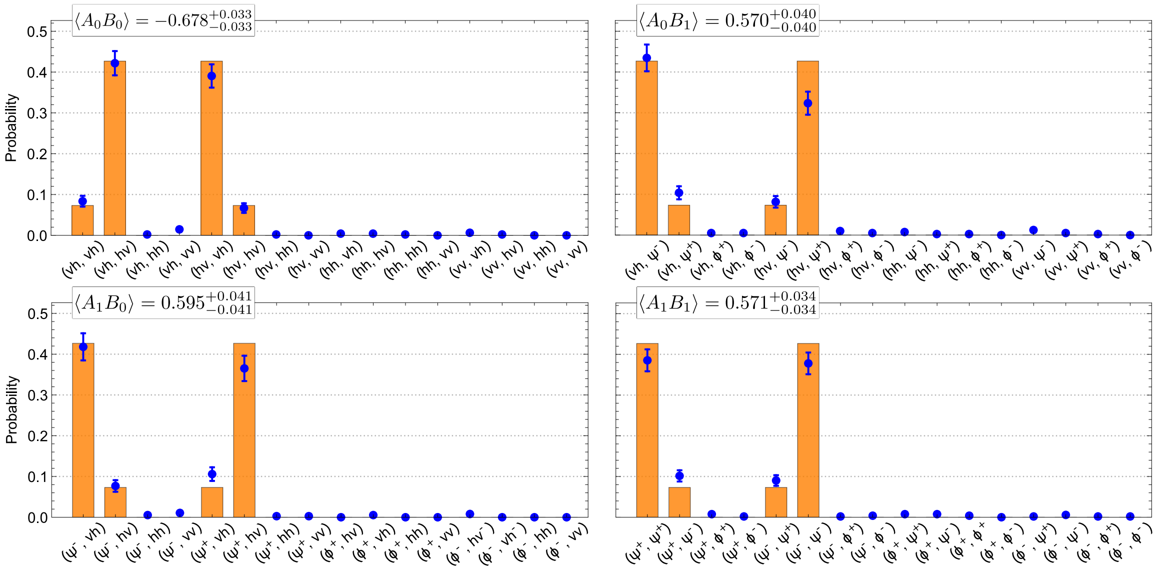

Fig. S2. Full experimental data. The full experimental set of probabilities for the 64 settings is shown. The horizontal axis in each of the four plots indicates the eigenstates (φ_A, φ_B) on which the experimental state shared by Alice and Bob in Eq.(11) is projected, where φ_A corresponds to Alice's projection in the two modes a and α, φ_B instead represents Bob's projection in modes b and β. For each setting, the number of 6-photon coincidences is recorded and normalised to obtain the relative probabilities as shown in the vertical axis.

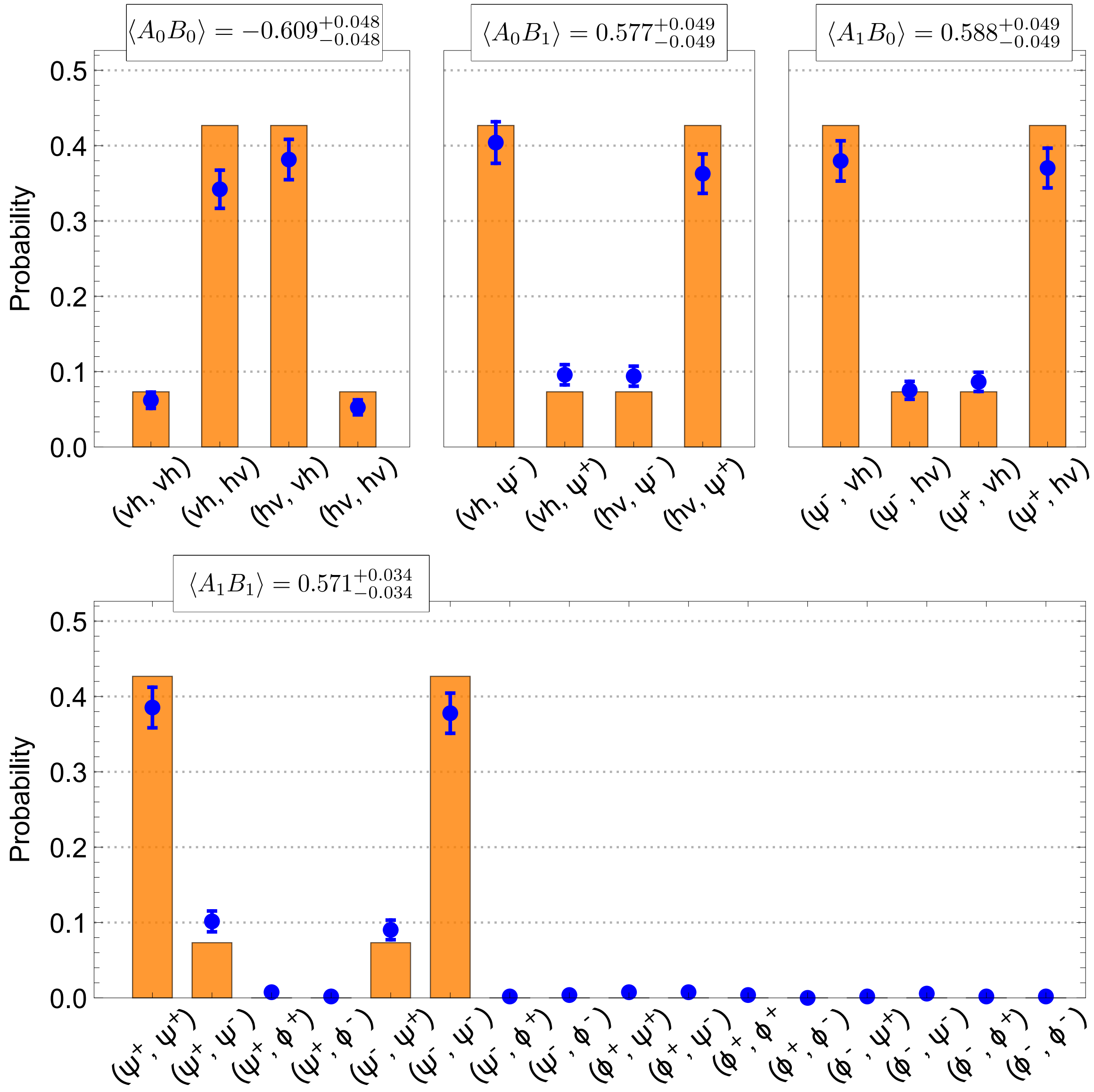

Fig. S3. Alternative protocol experimental data. The experimental probabilities obtained with the alternative definition of A_0 and B_0, Eq. (S1), are shown. $\langle A_1 B_1\rangle$, in the bottom panel, is left unchanged by the new definition thus the data shown here as well as the average value for this couple of observables, is the same as in Figs.(3) and S2. $\langle A_0B_0\rangle$, $\langle A_0B_1\rangle$ and $\langle A_1B_0\rangle$ shown in the top panels are instead measured according with the new protocol as explained in the Supplementary materials. In this case, only 6-photon coincidences for the non-zero terms, labelled in the horizontal axis, are recorded and normalised with the sum of all the coincidences recorded for $\langle A_1B_1\rangle$.
